# Curcumin Inhibits Glyoxalase 1—A Possible Link to Its Anti-Inflammatory and Anti-Tumor Activity

**DOI:** 10.1371/journal.pone.0003508

**Published:** 2008-10-23

**Authors:** Thore Santel, Gabi Pflug, Nasr Y. A. Hemdan, Angelika Schäfer, Marcus Hollenbach, Martin Buchold, Anja Hintersdorf, Inge Lindner, Andreas Otto, Marina Bigl, Ilka Oerlecke, Antje Hutschenreuter, Ulrich Sack, Klaus Huse, Marco Groth, Claudia Birkemeyer, Wolfgang Schellenberger, Rolf Gebhardt, Mathias Platzer, Thomas Weiss, Mookambeswaran A. Vijayalakshmi, Monika Krüger, Gerd Birkenmeier

**Affiliations:** 1 Institute of Biochemistry, University of Leipzig, Leipzig, Germany; 2 Frauenhofer Institute for Cell Therapy and Immunology IZI, Leipzig, Germany; 3 Institute of Clinical Immunology and Transfusion Medicine, Leipzig, Germany; 4 Department of Zoology, Faculty of Science, University of Alexandria, Moharram Bay, Alexandria, Egypt; 5 Leibniz Institute for Age Research-Fritz Lipmann Institute, Jena, Germany; 6 Institute of Analytical Chemistry, Leipzig, Germany; 7 Center for Liver Cell Research and Department of Surgery, University of Regensburg Hospital, Regensburg, Germany; 8 CBST; VIT University, TamilNadu, India; 9 Institute of Bacteriology and Mycology, Veterinary Faculty, Leipzig, Germany; AgroParisTech, France

## Abstract

**Background:**

Glyoxalases (Glo1 and Glo2) are involved in the glycolytic pathway by detoxifying the reactive methylglyoxal (MGO) into D-lactate in a two-step reaction using glutathione (GSH) as cofactor. Inhibitors of glyoxalases are considered as anti-inflammatory and anti-carcinogenic agents. The recent finding that various polyphenols modulate Glo1 activity has prompted us to assess curcumin's potency as an Glo1 inhibitor.

**Methodology/Principal Findings:**

Cultures of whole blood cells and tumor cell lines (PC-3, JIM-1, MDA-MD 231 and 1321N1) were set up to investigate the effect of selected polyphenols, including curcumin, on the LPS-induced cytokine production (cytometric bead-based array), cell proliferation (WST-1 assay), cytosolic Glo1 and Glo2 enzymatic activity, apoptosis/necrosis (annexin V-FITC/propidium iodide staining; flow cytometric analysis) as well as GSH and ATP content. Results of enzyme kinetics revealed that curcumin, compared to the polyphenols quercetin, myricetin, kaempferol, luteolin and rutin, elicited a stronger competitive inhibitory effect on Glo1 (K_i_ = 5.1±1.4 µM). Applying a whole blood assay, IC_50_ values of pro-inflammatory cytokine release (TNF-α, IL-6, IL-8, IL-1β) were found to be positively correlated with the K_i_-values of the aforementioned polyphenols. Moreover, whereas curcumin was found to hamper the growth of breast cancer (JIMT-1, MDA-MB-231), prostate cancer PC-3 and brain astrocytoma 1321N1 cells, no effect on growth or vitality of human primary hepatocytes was elucidated. Curcumin decreased D-lactate release by tumor cells, another clue for inhibition of intracellular Glo1.

**Conclusions/Significance:**

The results described herein provide new insights into curcumin's biological activities as they indicate that inhibition of Glo1 by curcumin may result in non-tolerable levels of MGO and GSH, which, in turn, modulate various metabolic cellular pathways including depletion of cellular ATP and GSH content. This may account for curcumin's potency as an anti-inflammatory and anti-tumor agent. The findings support the use of curcumin as a potential therapeutic agent.

## Introduction

Curcumin (1,7-bis(4-hydroxy-3-methoxyphenyl)-1,6-heptadiene-3,5-dione) is a polyphenol derived from the plant *Curcuma longa*. The medical use of this plant has its roots in the Indian Ayurveda medicine for over 6000 years. Extensive research over the last decades has indicated that curcumin exhibits anti-inflammatory, anti-oxidant, anti-viral and anti-infectious activities [1], [2]. Furthermore, in various animal models, curcumin was found to suppress symptoms associated with type II diabetes [3] rheumatoid arthritis [4], Alzheimer disease [5], promoted wound healing [6] and was protective in an animal model of sepsis [7]. These effects seem to be linked to the anti-inflammatory effect of curcumin that, in turn, may be attributed to its ability to inhibit cyclooxygenase-2, lipoxygenase and inducible nitric oxide synthase (iNOS) [8]. Recently, the involvement of the peroxisome proliferator-activated receptor-gamma has been shown [9].

Curcumin such as other polyphenols is as strong anti-oxidant [10]. It significantly decreases lipid peroxidation, regulates anti-oxidant enzymes and scavenges hyperglycemia-induced reactive oxygen species (ROS) [11]. Oxidative stress and inflammation are closely associated with tumor growth [12]. Therefore, it is not surprising that curcumin possesses anticancer effects by blocking different stages of the tumor development [13]. The low incidence of colon cancer among Indians has been attributed to the use of curcumin in cooking in addition to other dietary compounds [14]. However, a possible dominance of certain genetic prerequisites in these populations can not be excluded.

Anti-proliferative effects of curcumin are suggested to be mediated by induction of apoptosis in a mitochondria-dependent manner via caspase-3 activation [15]. In addition, curcumin could affect cellular proliferation by modulating of various cell signaling pathways including NF-κB, growth factor receptor kinases and mitogen-activated protein kinases [16]. Although, the multiple biological functions of curcumin have been described, the prime driving mechanism underlying its action remains unknown [17].

In the present study we provide evidence that the diverse actions of curcumin may be mediated by inhibiting cellular glyoxalases. Glyoxalases are involved in the detoxification of the reactive MGO formed para-metabolically and para-enzymatically from triosephosphates when glucose is degraded. Glo1 catalyzes the conversion of cytotoxic MGO to nontoxic hemithioacetal using GSH as cofactor. This enzyme is ubiquitously expressed in all mammalian cells and is suggested to be involved in cellular aging and cell death [18].

Inhibitors of Glo1 have long been sought as possible anticancer agents. High affinity inhibitors were prepared as GSH analogues such as S-p-bromobenzylglutathione and were shown to display anti-proliferative action [19]. However, the ubiquity of GSH and its involvement in vital cellular reactions may limit the use of native GSH analogues as inhibitors. We found that curcumin acts as a strong inhibitor of Glo1, causes depletion of cellular ATP and GSH and thus has a potential impact at cellular metabolism predominantly in cells whose energy gain relies on the glycolytic pathway. Inhibition of Glo1 seems to be an important route by which this dietary agent may exert its diverse biological effects.

## Results

### Curcumin inhibits glyoxalase 1 with high affinity

**Figure 1:**
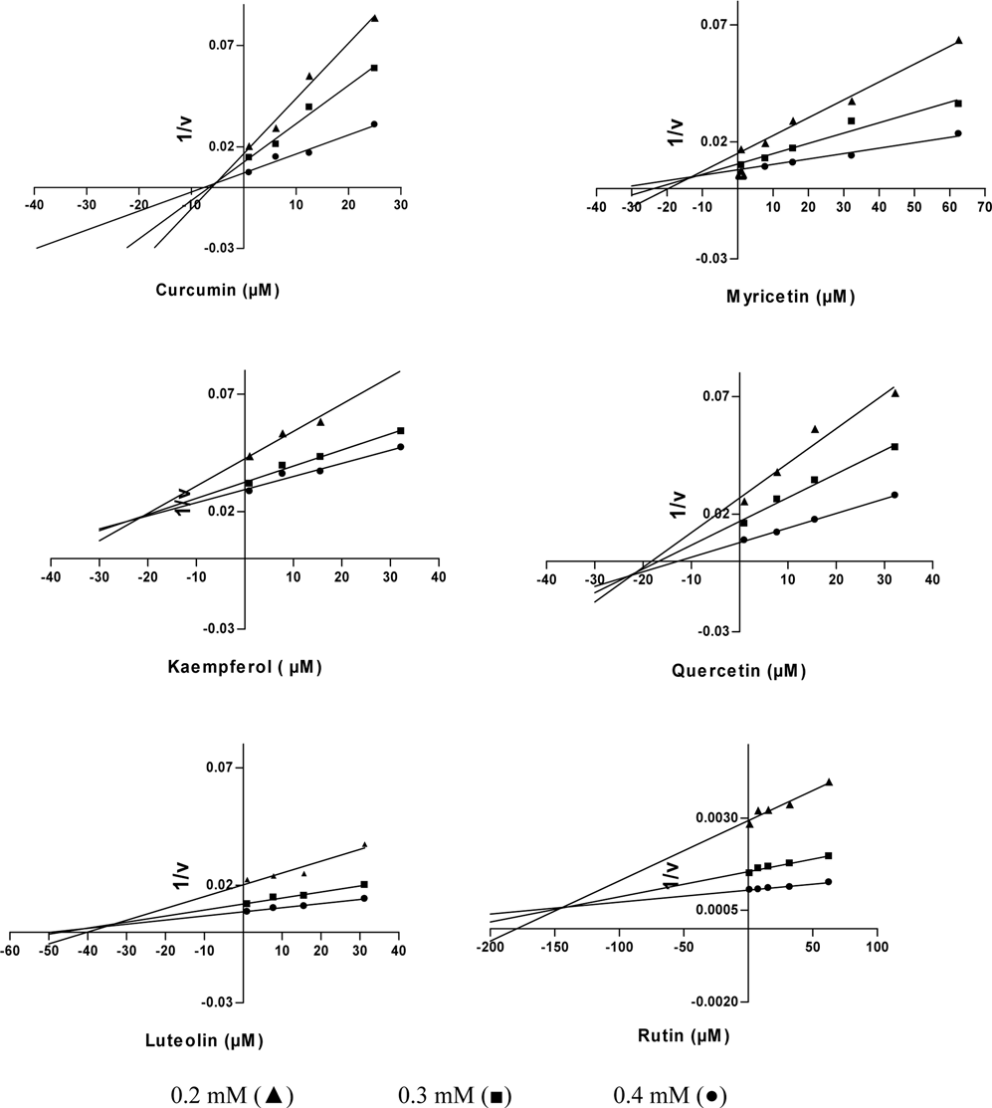
Inhibition of glyoxalase 1 activity by polyphenolic compounds. Purified Glo1 (70 mU) was incubated with increasing concentrations of different polyphenols at three substrate concentrations; and enzyme activity was recorded (n = 3). The inhibitor concentration was plotted vs. the reciprocal of enzyme activity (1/v) in Dixon plots.

Recently, some polyphenols such as quercetin, luteolin, myricetin, and kaempferol have been shown to inhibit Glo1 [20]–[22]. To see whether curcumin inhibits the enzyme similar to these dietary compounds, we analyzed the enzyme activity in the presence of increasing amounts of inhibitors at different concentrations of the substrates MGO and GSH. The Dixon plot analysis revealed that enzyme inhibition by curcumin was competitive with K_i_ = 5.1±1.4 µM (Fig. 1). Compared to curcumin, inhibition by other polyphenols turned out to be significantly lower and yielded Ki-values of 23±4.5 µM, 13±2.9 µM, 21±3.8 µM, 35±6.3 µM, 140±15 µM for quercetin, myricetin, kaempferol, luteolin and rutin, respectively.

### Curcumin exerts an anti-inflammatory effect on LPS-stimulated blood cells

**Figure 2:**
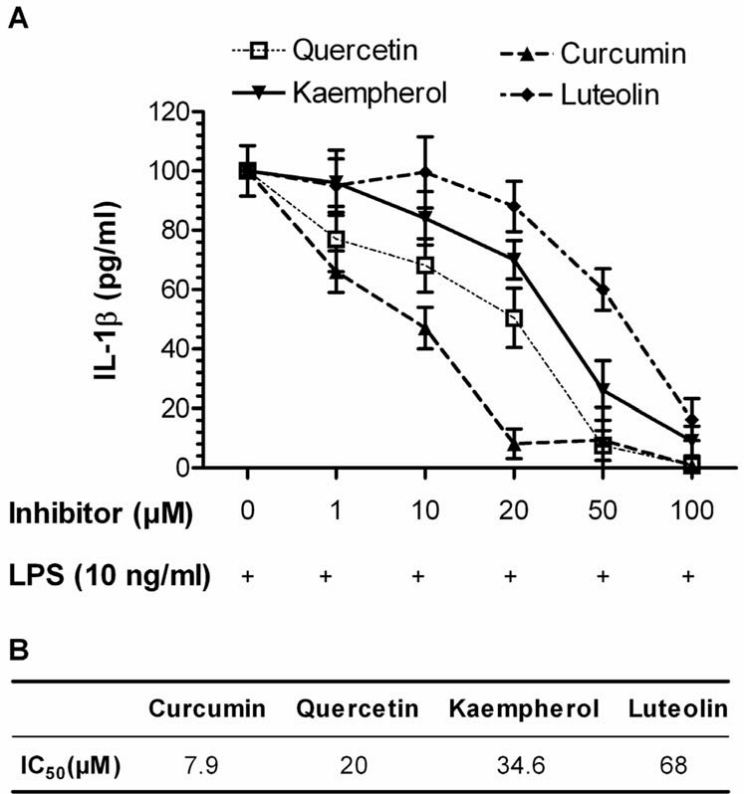
Effect of polyphenols on IL-1β release from LPS-stimulated blood cells. Heparinized whole blood was stimulated by LPS in the absence or presence of polyphenols at increasing concentrations and incubated for 6 h at 37°C with 5% CO_2_. (A) Released IL-1β as measured in cell supernatants. Samples without additives but LPS were set at 100%. (B) The calculated LD_50_ values of the respective polyphenols. Data represent mean±S.D. of independent experiments (n = 6).

Polyphenols are known for their anti-inflammatory effects on immune cells. To compare the effect of curcumin with other polyphenols displaying flavonoid structure, we applied whole blood assays whereby monocytes were stimulated with LPS at 37°C/5% CO_2_ and incubated for 6 h (Fig. 2). LPS-stimulated blood cells release pro-inflammatory cytokines such as TNF-α, IL-6, IL-1β and IL-8. Upon addition of curcumin and other selected flavonoids at increasing concentrations to the cultured cells, we observed a strong suppression of the pro-inflammatory cytokine release as exemplified by IL-1β (Fig. 2). The results of the current study rank curcumin at the top of the investigated polyphenols with respect to inhibition of pro-inflammatory cytokine production. IC_50_ values of 7.9 µM, 20 µM, 34.6 µM and 68 µM have been estimated for curcumin, quercetin, kaempferol, and luteolin, respectively. None of these compounds was found to induce IL-1β release in the absence of LPS. Moreover, at concentrations above 200 µM, these compounds induced red blood cell lysis. A strong positive correlation was found between the anti-inflammatory activity (IC_50_) and the *in vitro* K_i_-values of curcumin, quercetin, kaempferol and luteolin (Spearman's R = 0.90) indicating that Glo1 inhibition may be a possible mechanism to explain the anti-inflammatory effects of these polyphenols.

### Curcumin inhibits growth of cancer cells via targeting glyoxalase 1

**Figure 3:**
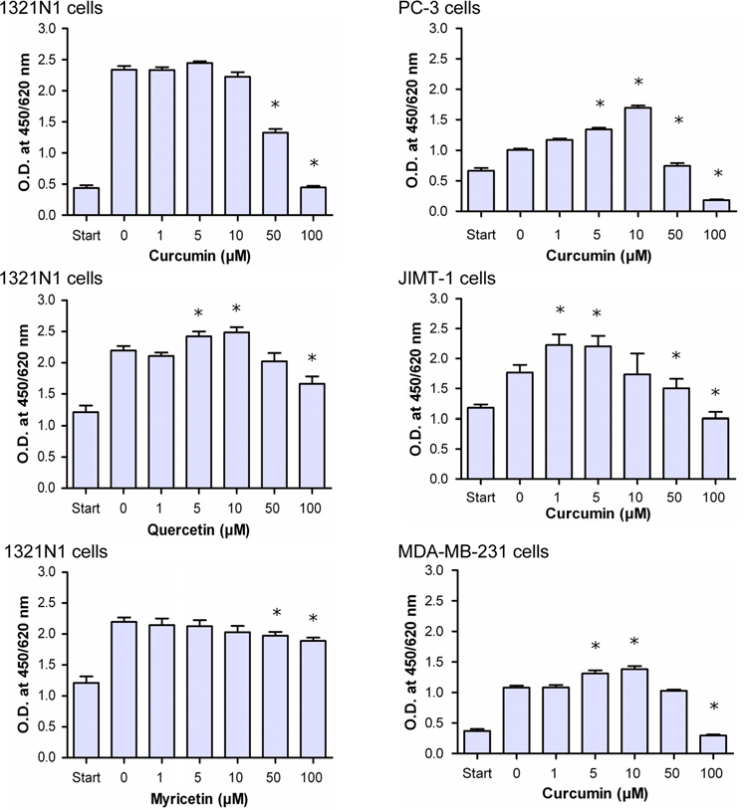
Growth inhibition of different tumor cell lines by polyphenols. Tumor cells (5000 cells/well) were seeded (start) and cultured at 37°C/5% CO_2_ in the presence of increasing concentrations of curcumin or the flavonoids quercetin or myricetin. Following 24-h incubation, cell proliferation was evaluated using the WST-1 assay.

It is known that inhibitors of Glo1, structurally related to GSH, have anti-proliferative properties [19]. To study the action of curcumin on cell growth, we incubated different tumor cells with increasing concentrations of curcumin for 24 h and measured changes in cell proliferation applying WST-1 assay (Fig. 3). Curcumin effectively inhibited the growth of different cancer cell lines derived from prostate cancer (PC-3), breast cancer (MDA-MB-231, JIMT-1), and brain astrocytoma (1321N1). Curcumin-treated cells manifested a dose-dependent reduction in cell proliferation (Fig. 3A). Obviously, the cellular activity of curcumin is biphasic. At low concentrations, it is stimulatory rather than inhibitory, especially in the range between 1 µM and 10 µM. This effect was observed predominantly in prostate and breast cancer cells and was absent in astrocytoma cells. However, strong anti-proliferative effects were observed at concentrations above 50 µM for all cancer cells tested. Not only did curcumin inhibit cell growth as seen for 1321N1, MDA-MB-231 and JIMT-1 cells, but it also exerted even a toxic effect at 100 µM on PC-3 cells. In this case, the normal cellular morphology got lost indicating necrotic cell death. Comparable to curcumin, both of quercetin and myricetin, which also inhibited Glo1 activity, were less anti-proliferative to 1321N1 cells. This indicates that the growth suppressing effect of the studied polyphenols may be related to the K_i_-values for Glo1 inhibition as shown in figure 1.

**Figure 4:**
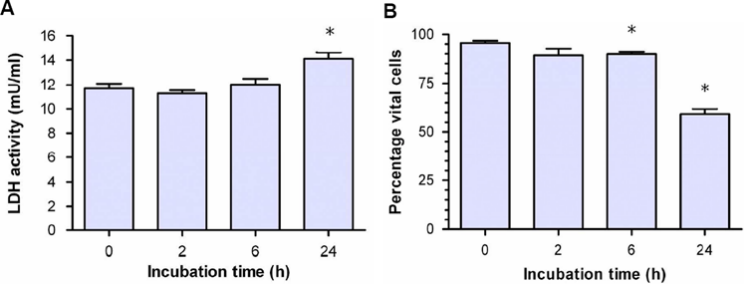
Release of L-lactate dehydrogenase (L-LDH) and vitality of 1321N1 cells upon incubation with 50 µM curcumin. Tumor cells were cultured and treated as mentioned in Fig. 3. (A) LDH activity was measured in cell supernatants. (B) Cell vitality as assayed by the trypan blue exclusion test. Data represent the mean±S.D. of independent experiments (n = 6).

Following 6-h incubation of 1321N1 cells with curcumin (50 µM), cell shrinking and chromatin condensation accompanied by a massive loss of cytoplasm was observed (data not shown). This may be indicative to metabolically depressed tumor cells. Surprisingly, cell membranes integrity at this stage was apparently not altered as indicated by L-LDH release that was not significantly increased (Fig. 4A), as well as by the percentage of vital cells measured by trypan blue exclusion that was only slightly diminished (89.5%±2.5% vs. 95.6%±2.5%) (Fig. 4B). However, longer incubation (24-h) increased the LDH release and significantly reduced the number of vital cells to 60%±6.1% relative to the control.

**Figure 5:**
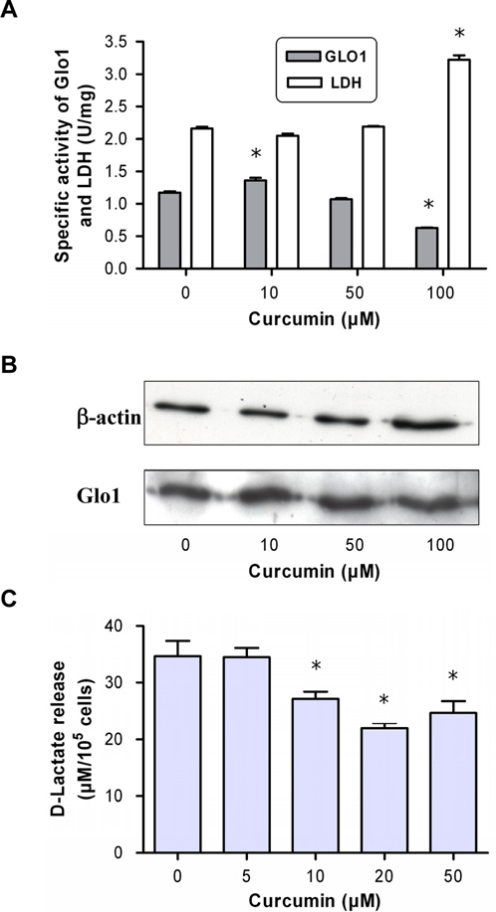
Cytosolic activity and protein content of glyoxalase 1 upon treatment of JIMT-1 cells with curcumin. (A) JIMT-1 cells were treated with curcumin at 37°C and 5% CO_2_ for 24 h. Cells were harvested and the specific activity of Glo1 and LDH was determined in the cytosolic extract. (B) Semi-quantitative analysis of protein content as performed by Western blot using specific antibodies against β-actin and Glo1. The blot was developed by chemoluminescense detection. (C) D-lactate release as determined in supernatants of astrocytoma 1321N1 cells following 24-h incubation with curcumin. Data represent the mean±S.D. of independent experiments (n = 6).

To proof that inhibition of the Glo1activity accounts for cellular effects, we exposed JIMT-1 breast carcinoma cells to curcumin for 24 h. After cell harvesting and solubilisation, we found that specific Glo1 activity in treated cells was lower than in non-treated cells and decreased dose-dependently (Fig. 5A). As a control, we measured the LDH activity, another cytosolic enzyme, which we found to be increased at higher curcumin concentrations. This indicates the very selective effect of curcumin at the para-glycolytic enzyme Glo1. Again, a small augmentation of Glo1 activity was observed at 10 µM curcumin which may reflect the findings shown in figure 3. Estimating the protein level, Western blot analysis revealed no alteration of Glo1 content (Fig. 5B). This substantiates the assumption that curcumin acts as an enzyme inhibitor rather than through modulating the expression or degradation of Glo1. We found similar effects with other tumor cells. To corroborate the results, we measured the concentration of D-lactate in the medium of cultured 1321N1 cells incubated with increasing concentrations of curcumin for 24 h. D-lactate is produced in the MGO pathway as the end-product of the Glo2 enzymatic reaction. Inhibition of glyoxalases is known to result in a decrease in D-lactate release. Our results demonstrated that D-lactate decreased from 34.6±6 µM in the control cells to 22±1.9 µM at 20 µM curcumin.(Fig. 5C). This indicates that cytosolic Glo1 is inhibited by curcumin and thereby may result in accumulation of MGO.

**Figure 6:**
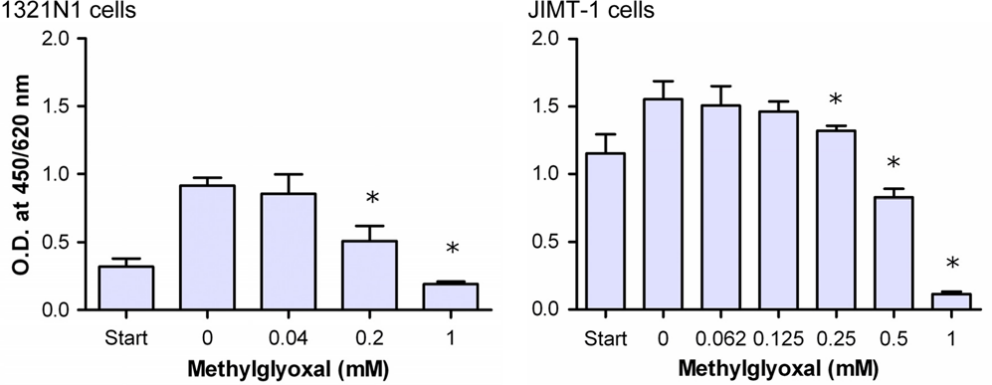
Effect of methylglyoxal on proliferation of astrocytoma and breast cancer cells. Astrocytoma 1321N1 (5000 cells/well) and breast cancer cells JIMT-1 were seeded (start) and cultured in the absence or presence of increasing concentrations of MGO for 24 h. Cell vitality was recorded by WST-1 assay. Data represent the mean±S.D. of independent experiments (n = 12).

Because MGO rapidly forms hemithioacetal with GSH, elevated MGO will, in turn, deplete cellular GSH levels especially at low expression level of Glo2. Therefore, we investigated whether MGO may, at least partially, account for the anti-proliferative effect of curcumin by treating 1321N1 and JIMT-1cells with increasing concentrations of the reactive aldehyde (Fig. 6). The results disclose the anti-proliferative properties of MGO. Because MGO exerts its effect predominantly intracellular, we expected that the concentration of MGO that reaches the cell is much lower than what was added to the medium. This means that MGO rapidly reacts with amino and sulfhydryl groups of proteins abundantly present in the culture medium (10% fetal calf serum). In this regard, it has been reported that the intracellular concentrations of MGO in normal growing cells can be elevated up to 300 µM [23].

**Figure 7:**
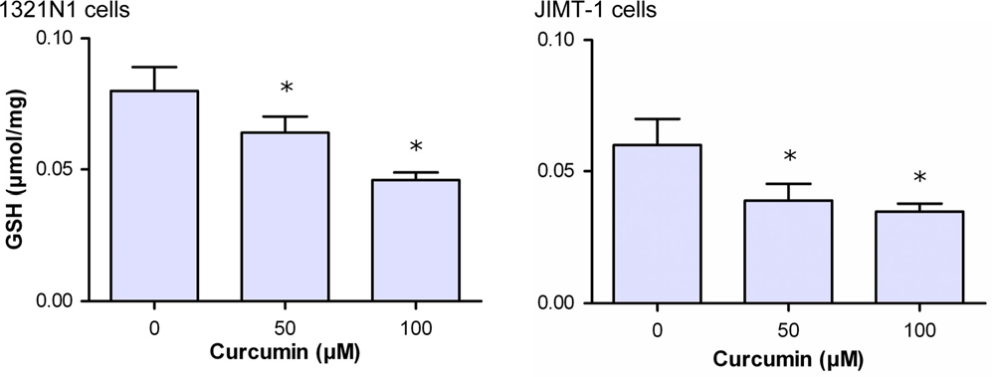
Effect of curcumin on glutathione (GSH) level of 1321 N1 and JIMT-1 cells. Astrocytoma 1321N1 (A) and JIMT-1 (B) cells were cultured for 24 h at 37°C and 5% CO_2_ in the absence or presence of curcumin. Cells were harvested and GSH concentration was determined in the cytosolic extract. Data represent the mean±S.D. of independent experiments (n = 6).

As shown in figure 7, curcumin-treated 1321N1 and JIMT-1 cells revealed decreased GSH levels. GSH was diminished by approximately 20% in cells treated with 50 µM curcumin, a concentration which yields approximately 30% inhibition of cell proliferation as presented in figure 3. This may be linked to the increasingly formed hemithioacetal that might trap GSH. This trapping can be magnified in case of a low Glo2 activity, an enzyme that hydrolyzes the S-lactoylglutathione, the end product of Glo1, to release D-lactate and to regenerate GSH. Therefore, we measured the Glo2 activity in cytosolic cell extracts (Table 1). The results demonstrated between 10- and 70-fold decrease in specific activity of Glo2 compared to Glo1 in different tumor cell lines. Thus, the low expression of Glo2 is suggested to impair the regeneration of GSH. Obviously, this could add additional effects to the GSH depletion.

**Table 1:** Specific activity of Glo1^1^, Glo2 and GSH^2^ content in various tumor cells.

Cell type	Glo1 (U/mg protein)	Glo2 (U/mg protein)	GSH (µmol/mg protein)
**Brain astrocytoma 1321N1**	0.9±0.08	0.02±0.004	0.08±0.005
**Breast cancer MDA-MD-231**	0.4±0.04	0.03±0.008	0.03±0.003
**Prostate cancer PC-3**	1.4±0.09	0.02±0.009	0.05±0.009
**Breast cancer JIMT-1**	1.17±0.02	0.02±0.01	0.06±0.01

Cytosolic extracts were prepared from cultured tumor cells and specific activity of enzymes and GSH was determined as described in Materials and Methods. The values represent means±S.D. determined in triplicates (n = 6).

1 glyoxalase 1 & glyoxalase 2.

2 glutathione.

### Curcumin leads to metabolic depletion of ATP

**Figure 8:**
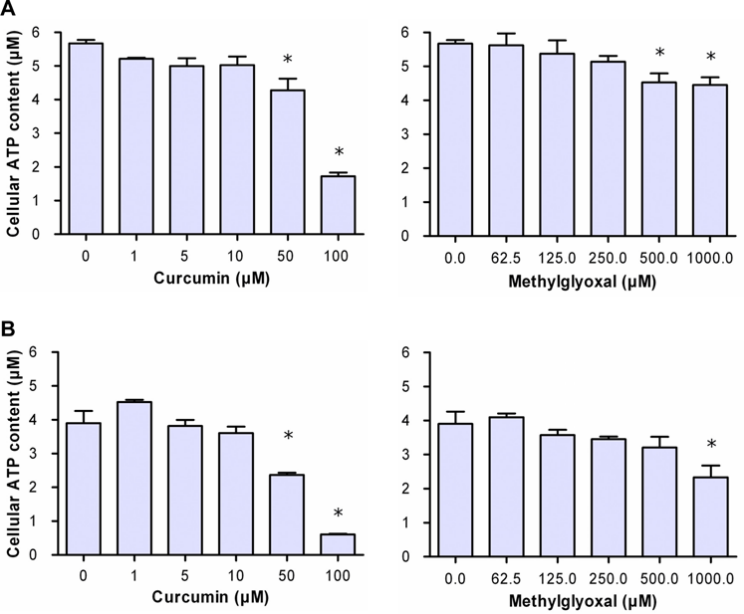
Effect of curcumin and methylglyoxal (MGO) at the ATP level in tumor cells. Astrocytoma 1321N1 cells (A) and JIMT-1cells (B) were seeded (5000 cells/well) and cultured in the presence of curcumin (0–100 µM) or MGO (0–1000 µM) for 6 h. Cellular ATP content was determined using the CellTiter-Glo® Luminescent Cell Viability Assay. Data represent the mean±S.D. of independent experiments (n = 12).

It has been shown that MGO affects energy metabolism [24]. If curcumin-induced Glo1 inhibition leads to accumulation of MGO, both of curcumin and MGO are expected to have similar effects at metabolic activity of tumor cells. Therefore, we analyzed the ATP-content of 1321N1 and JIMT-1 cells treated with various concentrations of curcumin and MGO. As shown in figure 8, curcumin decreased cellular ATP in a concentration-dependent manner and MGO disclosed comparable effects.

### Curcumin is cytotoxic to cancer cells but not to primary human hepatocytes

**Figure 9:**
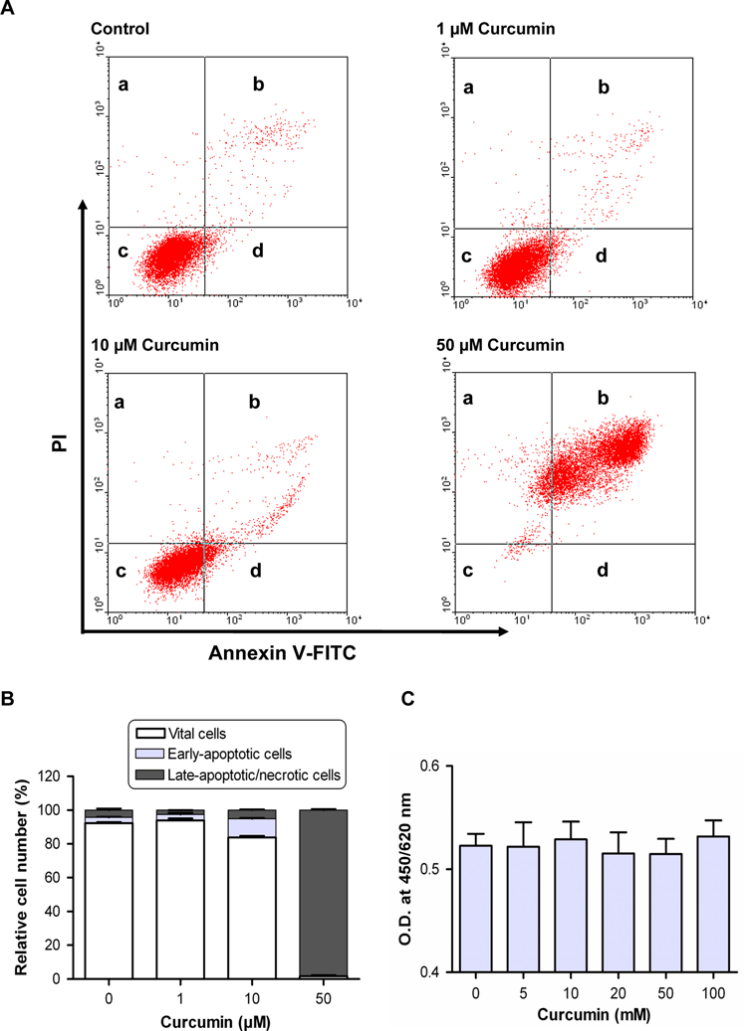
Effect of curcumin on apoptosis/necrosis of tumor and normal cells. Apoptosis/necrosis was evaluated using annexin V-5-fluorescein isothiocyanate (FITC)/propidium iodine (PI) staining followed by flow cytometric analysis. (A) Representative plots of showing annexin V-FITC/PI staining of Astrocytoma 1321N1 cells cultured in the presence of curcumin. The proportion of dead cells (a: annexin V-FITC^−^/PI^+^), late apoptotic/necrotic cells (b: annexin V-FITC^+^/PI^+^), non-apoptotic cells (c: annexin V-FITC^−^/PI^−^) and early apoptotic cells (d: annexin V-FITC^+^/PI^−^). (B) Results of 3 independent experiments. (C) Vitality response of primary human hepatocytes to 24-h incubation with curcumin as assessed by WST-1 test. Data represent the mean±S.D. of three independent experiments.

Increased MGO [25], depletion of GSH [26] and ATP [27] are known to initiate apoptotic cell death. We questioned whether curcumin inhibits tumor cell growth by induction of apoptosis. Therefore, 1321N1 cells were treated with increasing amounts of curcumin and analyzed for annexin V-5-fluorescein isothiocyanate (FITC)/propidium iodide (PI) binding (Fig. 9A and 9B).

The results show that curcumin induced apoptosis in 1321N1 cells. Curcumin at a concentration of 10 µM was found to induce early apoptosis of 11.0% of cells characterized by the intactness of membrane, affinity towards annexin V-FITC and devoid of PI staining. At higher concentration of curcumin (50 µM), the number of late apoptotic/necrotic cells increased up to 98.2%. This accords the results shown in figure 4.

Primary human hepatocytes, on the other hand, were not affected by curcumin as shown by the lack of any change in the cell vitality in the presence of increasing concentrations of curcumin (Fig. 9C).

## Discussion

### Anti-proliferative and anti-inflammatory activity of curcumin

Curcumin possesses a wide range of pharmacological properties including anti-inflammatory, anti-infectious, and anti-carcinogenic activities [28]. A number of different targets have been proposed to mediate these different effects which include transcription factors, enzymes, cytokines and cell receptors [1], [13]. In the present work we have added a further target of curcumin, the Glo1. Notwithstanding the numerous studies, Glo1 has never been reported or suggested to be a binding target of curcumin.

Inhibitors of glyoxalases have long been sought as possible anti-cancer drugs. In this sense, analogues of GSH such as S-*p*-bromobenzylglutathione have been prepared and used to inhibit the growth of cancer cells [19]. However, the use of high affinity glutathione-based inhibitors has many drawbacks due to interference with many other GSH-dependent enzymes *in vivo*.

Recently, natural inhibitors of Glo1 have been selected from flavonoids, polyphenolic compounds found in many plants [22]. This prompted us to test whether the polyphenol curcumin exerts similar properties. We found that curcumin showed even a stronger inhibitory effect on Glo1 than the flavonoids quercetin, myricetin, kaempferol, luteolin and rutin. Its inhibitory activity expressed by the K_i_ value was in the range of that of S-*p*-bromobenzylglutathione known to be the strongest inhibitor of Glo1 [22].

Growth inhibition curve of cancer cells treated with curcumin showed a biphasic feature. At low concentrations, curcumin was found to stimulate cell proliferation rather than being inhibitory. The reason for this biphasic action is currently not known, but it may be related to the polyvalent activity of curcumin, so that different cellular pathways are activated in a concentration-dependent manner. Such a distinct dose-related behavior was already observed in case of stimulated mouse macrophages treated with curcumin [29]. At high concentrations, curcumin was found to act probably via inactivation of NF-κB compared to modulation of the heme oxygenase expression at low concentrations.

Similarly, curcumin was found to act as an anti-oxidant at low doses, whereas at higher doses the pro-oxidant properties prevailed, a virtue that may be of high importance in cell apoptosis and treatment of cancer cells [30]. In addition, the down-regulation of pro-inflammatory cytokines is most likely mediated by inactivation of the transcription factor NF-κB signaling system although the precise mechanism is not known [31]. No chemical modification of the NF-κB signaling pathway by curcumin was found and the site of intervention was supposed to precede the phosphorylation steps.

We could imply that curcumin had a strong impact at the highly proliferative and invasive tumor cells such as 1321N1, PC-3 and MDA-MB-231. Currently, there are no drugs for successful treatment of human astrocytoma and glioma tumors. This might be of interest in the light of the finding that curcumin is capable to cross the blood-brain-barrier [5].

This would increase the list of therapeutic application of curcumin comprehensively described earlier [32].

The striking differences found between the IC_50_ values for cytokine suppression compared to values for the anti-proliferative activity of curcumin let us assume that this polyphenol possesses an anti-inflammatory potency at low concentrations, whereas higher concentrations are required to mediate an anti-proliferative effect. This can be explained by the different expression of Glo1 in mononuclear blood cells compared to tumor cells. Glo1 is highly expressed in cancerous cells to compensate for the permanent formation of MGO due to the high glycolytic rate. The specific activity of Glo1 in mononuclear blood cells was approximately 1/10 compared to tumor cells (unpublished data). Thus, lower concentrations of Glo1 inhibitors are required to mediate cellular effects.

### Proposed mechanism of curcumin action

The expression of glyoxalases is normally adjusted to the flux of glucose through the glycolytic pathway. Many cancer cells gain their energy mainly by oxidation of glucose to lactate showing a high aerobic glycolysis [33]. To compensate the low ATP gain via glycolysis, these cells must increase their glycolytic flux several folds. Consequently, this increases the level of MGO to toxic concentrations for the respective cells [24]. Therefore, most cancer cells show increased expression of Glo1 [34].

The key events in cellular metabolism upon glyoxalase inhibition are the elevated levels of MGO and depletion of GSH. Both alterations will have profound effects on regulatory processes and on cellular bioenergetics. There are reports showing that MGO is toxic to cells by depleting ATP, modulation of mitochondrial membrane potential, induction of apoptosis and ROS-production [24], [35]. MGO is capable to modify the function of very distinct proteins, e.g. activation of transcription factors in yeast [36], distinct covalent modifications of proteins like Hsp27 [37] or modulation of enzyme activity of GAPDH [38]. Results of a recent paper supported our hypothesis in the broadest sense that MGO, by reacting with Cys 38 of NF-κB p65, inhibits its binding to DNA [39]. These data coincide with the observed effects of curcumin at the mitochondrial membrane permeability and inhibition of ATP synthesis [40], as well as suppressed activation of NF-κB. However, the specific target within this signaling pathway has not been identified yet [41]. NF-κB activation enables anti-apoptotic genes to promote carcinogenesis and induces inflammation [42]. Therefore, we suggest that the anti-proliferative and ant-inflammatory effect of curcumin might be at least partially mediated by inhibition of Glo1.

As far as GSH is concerned, increased levels of MGO may cause cellular depletion of GSH due to hemithioacetal formation. Low levels of GSH have been found to decrease resistance of cancer cells to chemotherapeutic drugs [43], boost cell apoptosis [44] and mediate ROS-induced mitochondrial damage [45]. Without knowing the precise site of action, curcumin treatment was associated with decreased intracellular GSH which may account for its pro-apoptotic effects [46] but is inconsistent with the observed anti-oxidant properties of curcumin [47]. Recently, it has been convincingly shown that a number of flavonoids can induce GSH depletion in human tumor cells. However, the mechanism how polyphenols can drive GSH depletion has not been published yet [48]. The observed decrease of intracellular GSH and ATP content of tumor cells may cooperate in mediating toxic effects on cells at higher concentrations of curcumin. As far as ATP depletion is concerned, it can currently not be dissipated whether this may be caused by direct inhibition of glycolysis or by toxic effects of MGO or curcumin on mitochondria.

**Figure 10:**
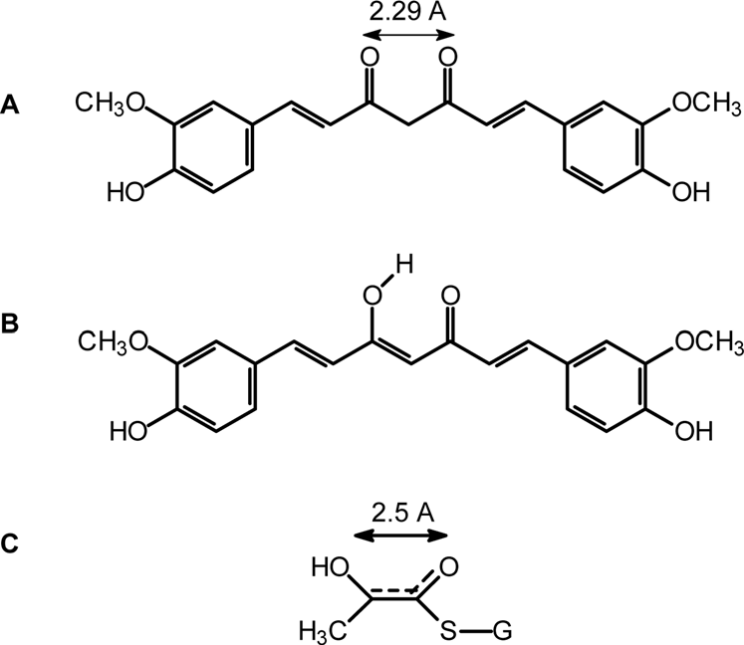
Keto-Enol Tautomeric forms of curcumin and transition-state of glutathione (GSH) and methylglyoxal (MGO). (A) (*1E,6E*)-1,7-bis(4-hydroxy-3-methoxyphenyl)hepta-1,6-diene-3,5-dione. (B) (*1E,4Z,6E*)-5-hydroxy-1,7-bis(4-hydroxy-3-methoxyphenyl)hepta-1,4,6-triene-3-one. (C) Transition-state compound of reaction between GSH and MGO.

Inhibition of Glo1 by compounds unrelated to GSH has been described recently for flavonoids and indomethacin [22], [49]. Selected from flavonoids, Takasawa *et al.* [22] proposed that compounds possessing a plane configuration of a ketone and a hydroxy group arranged in a distance of 2.8 A may mimic the enediolate intermediate that yields along the reaction pathway of Glo1 (Fig. 10). As curcumin is endowed with similar structural elements (keto-enol forms), it is conceivable that this pharmacophore directly interacts with the active site of Glo1. Curcumin may exist at equilibrium between the diketo and keto-enol forms; the latter is strongly favored by intramolecular H-bonding. In this line, it has been suggested that these structural elements appear to be important for its anti-tumor activity [50]. Recently, we could evidenced that compounds harboring “dicarbonyl” groups such as ethyl pyruvate possess an anti-inflammatory potency and suppress the expression of immune receptors on human macrophages via targeting human Glo1 [51].

It has been reported that curcumin can directly react with SH groups of glutathione and cysteine residues in proteins [52]. However, it is not conceivable that this may affect the cellular GSH content because the intracellular concentration of curcumin is expected to be too low compared to that of GSH.

Among the different known targets of curcumin, only Glo1 bridges energy metabolism, especially glycolysis, to inhibition of cell growth. That may be the reason why Glo1 is critical for cell survival [53] and that Glo1 inhibitors may constitute potent chemotherapeutic agents [34]. In addition, it may also explain why curcumin is also active against inflammation, bacteria and protozoa [11], [54], [55]. Inflammation process is always accompanied by activation of glycolytic pathway in specific cells such as monocytes/macrophages or lymphocytes. Furthermore, bacteria as well as protozoa such as malaria parasites and trypanosome are mainly glucose-consuming organisms. As these organisms do not possess NF-κB pathway, one of the predicted targets of curcumin, it is likely that curcumin may act through glyoxalase inhibition, in addition to other proposed mechanisms [56].

In conclusion, the present study may designate the natural product curcumin as a strong competitive inhibitor of Glo1. This will increase our understanding of the anti-inflammatory and anti-proliferative effects of curcumin and may provide new insights into how curcumin exerts its anti-carcinogenic effect.

## Materials and Methods

### Materials

Curcumin, myricetin, luteolin, kaempferol and quercetin were obtained from Roth (Karlsruhe, Germany). 5,5′-Dithio-bis(2-nitrobenzoic acid) (DTNB), methylglyoxal, lipopolysaccharide (*E. coli* Serotype O111:B4), S-lactoylglutathione, propidium iodide, trypan blue, L-lactate, D-lactate, D-lactate dehydrogenase (D-LDH) (*Lactobacillus leichmannii*) and L-lactate dehydrogenase (L-LDH) (bovine heart), EDTA, glycerol, Triton X-100, 1,4-dithiothreitol (DTT), phenylmethanesulfonyl fluoride (PMSF), William Medium E and protease inhibitor cocktail were purchased from Sigma-Aldrich (Taufkirchen, Germany). RPMI 1640 medium was purchased from Biochrom (Berlin, Germany). Cell proliferation reagent WST-1 was obtained from Roche (Mannheim, Germany). BD™ CBA Human Inflammation Kit was purchased from BD Biosciences (Heidelberg, Germany). Annexin V-FITC was obtained from Immunotools (Marseille, France). Dulbecco's modified Eagle Medium (DMEM), RPMI Medium and fetal calf serum (FCS) were obtained from Invitrogen (Karlsruhe, Germany). Anti-human Glo1 monoclonal antibodies were purchased from BioMac (Leipzig, Germany). Tumor cell lines used are prostate cancer PC-3 (CRL-1435; ATCC), brain astrocytoma 1321N1 cells (86030102; ECACC) and human breast JIMT-1 (ACC 589; DSMZ) and MDA-MB-231 (HTB-26; ATCC) cancer cells.

### Application of curcumin

A stock solution of curcumin (50 mM) was prepared in DMSO. Just before adding to cultured cells, curcumin was diluted in culture medium as required keeping the final DMSO concentration at 1%.

### Whole blood assay

Whole blood assay was performed as previously described [57]. Accordingly, LPS (10 ng/ml), test substances and 200 µl heparinized human blood of healthy donors, obtained from the blood bank of the University of Leipzig, were co-incubated with serum-free medium (RPMI-SF, 1 ml final volume) in 24-well culture plates for 6 h at 37°C with 5% CO_2_. Samples were removed, centrifuged at 2000×g for 10 min and supernatants were stored at −20°C until assaying for inflammatory cytokines.

### Cytokine analysis using cytometric bead array

Assessment of cytokine levels in supernatants of human blood cell cultures was accomplished using Cytometric Bead Array (CBA™, BD Biosciences, San Jose, U.S.A). The procedure was carried out according to the manufacturer's instruction. Fluorescence measurement and analysis were performed using a FACSCalibur and the CellQuest software (BD Biosciences).

### Mammalian cell culture

Tumor cells were cultured in RPMI medium containing 10% fetal calf serum (RPMI-FCS) (PC-3, JIMT-1, MDA-MD 231) or in DMEM-FCS (astrocytoma 1321N1 cells) containing penicillin/streptomycin (100 U penicillin/ml; 100 µg streptomycin/ml) at 37°C/5% CO_2_. Cytosolic protein extracts of tumor cells were prepared using extraction buffer composed of 25 mM Tris, 2 mM EDTA, 10% Glycerol, 1% Triton ×100, 2 mM DTT, 1mM PMSF, pH. 7.0 containing 0.3% protease inhibitor cocktail. Protein content was determined according to Bradford [58]. Specific activity of enzymes analyzed in cell extracts was expressed as U/mg protein.

Primary human hepatocytes were isolated and cultivated in serum-free William Medium E [59]. Tissue samples from human liver resection were obtained from patients undergoing partial hepatectomy for metastatic liver tumor of colorectal cancer. Experimental procedures were performed according to the guidelines of the charitable state controlled foundation (Human Tissue and Cell Research), with the informed patient's consent [60] approved by the local ethical committee.

### Purification of glyoxalases

Glo1 was purified from human erythrocytes as described earlier [61]. Briefly, erythrocytes were treated with butanol/chloroform to denature hemoglobin followed by acetone and ammonium sulphate fractionation. Repeated affinity chromatography on S-hexylglutathione-Agarose followed by gel permeation chromatography yielded the enzyme; purity>90%.

### Glyoxalase activity measurement

The determination of Glo1 (E.C.4.4.1.5) activity has been described previously [61]. To evaluate the effect of curcumin and other polyphenols on Glo1 enzymatic activity, 2 mM GSH and 2 mM MGO were pre-incubated for 4 min, and then mixed with increasing concentrations of test substances together with 70 mU of the enzyme. The inhibition constant, K_i_ value of the inhibitors was evaluated by Dixon plot showing the reciprocal of enzyme velocity against the inhibitor concentration at variable concentrations of the substrate. Human Glo2 (E.C.3.1.2.6) was measured using the substrate S-lactoylglutathione as described earlier [62].

### LDH activity measurement

LDH activity was spectrophotometrically assayed in the cytosolic extract or in the of cell cultures by adding aliquots to 1 ml reaction buffer (50 mM Na_2_HPO_4_, pH 7.0, 0.15 mM NADH, and 0.3 mM pyruvate). Enzyme activity was recorded at 340 nm. It was ascertained that the enzyme activity was not affected by curcumin.

### Measurement of L-lactate and D-lactate

The determination of D-lactate and L-lactate in supernatants of astrocytoma 1321N1 cells was performed by an enzymatic method based on the oxidation of L-lactate and D-lactate to pyruvate by NAD in the presence of L-LDH or D-LDH[63].

### Apoptosis/necrosis measurement

Astrocytoma 1321N1 cells were treated with increasing amounts of curcumin for 24 h, washed with PBS and after trypsination, apoptosis/necrosis was evaluated using annexin V-FITC/propidium iodine (PI) staining as previously described [64]. Samples were analyzed by flow cytometry using a FACSCalibur and the CellQuest software (BD Biosciences, San Jose, USA). Laser excitation wavelength was set at 488 nm. The green signal from annexin V-FITC was measured at 525 nm and the red signal from PI was measured at 620 nm. Annexin V^−^/PI^−^ cells are viable; annexin V^+^/PI^−^ cells are in early apoptosis, whereas annexin V^+^/PI^+^ cells are necrotic or in late apoptosis are

### Glutathione determination

GSH was determined using DTNB method [65]. Briefly, cytosolic extract of tumor cells were precipitated by 5% sulfosalicylic acid neutralization, aliquots were mixed with DTNB and absorbance was read at 412 nm against a standard solution of GSH.

### Cell proliferation/vitality assays

Cell proliferation/vitality of tumor cells (96-well plates; 5000 cells/well) and primary human hepatocytes (24-well plates; 0.25×10^6^ cells/well) was assayed using the WST-1 assay according to manufacturer's instructions. Trypan blue exclusion was performed by staining cells with 0.4% aqueous trypan blue solution according to the manufacturer's instructions.

### ATP measurement

Cell ATP content was determined by means of the CellTiter-Glo® Luminescent Cell Viability Assay according to the manufacturer's instructions (Promega, Madison, USA). Briefly, tumor cells were cultured in 96-well plates (5000 cells/well) in the absence or presence of curcumin or MGO, respectively. After 6-h incubation, cells were mixed with test reagents and luminescence was read according to the manufacturer's instructions. A standard curve was prepared from ATP (1 µM to 10 nM) in medium.

### Western blot analysis

An amount of 20 µg to 40 µg of cytosolic extract was loaded to SDS-pore gradient gels (4 to 20%) and run under reducing conditions. Proteins were blotted to cellulose nitrate membranes (Whatman Schleicher & Schuell, Dassel, Germany) and Glo1 was detected by anti-Glo1 monoclonal antibodies (1 µg/ml) (BioMac, Leipzig, Germany) in combination with goat anti-mouse Ig-HRP (1∶1000) (Dako, Hamburg, Germany). For comparison, β-actin was analyzed using rabbit anti-β-actin Ig (1∶2000) (Acris, Hiddenhausen, Germany) in conjunction with HRP-labeled goat anti-rabbit Ig (Dianova, Hamburg, Germany). Band visualization was performed by chemiluminescence detection (Boehringer, Mannheim, Germany).

### Statistical analysis

Kinetic data (Fig. 1) were analyzed with the GraphPad Prism 3.03 (GraphPad Software Inc. San Dieago, CA) using the Marquardt-Levenberg method with simple weighting. A goodness-of-fit criterion (Akaike's information criterion, AIC) was used for falsification of incompatible inhibition models. It turned out that the considered inhibitors act mainly in a competitive mode. The quality of the fit was characterized by 95% confidence intervals of the estimated parameters and the total residual error. Spearman's rank correlation test was applied to evaluate the correlation between the IC_50_ values of pro-inflammatory cytokine release and the K_i_-values of various polyphenols. Data were presented as means±S.D. of at least three independent experiments. The enzyme inhibition by polyphenols (K_i_-values) are considered significant at p = 0.1 (Wilcoxon's rank-sum test). In all other tests, significance has been considered at values of p<0.05.
