# Correction: Curcumin Inhibits Glyoxalase 1—A Possible Link to Its Anti-Inflammatory and Anti-Tumor Activity

**DOI:** 10.1371/annotation/0eb2b9f3-1006-4dcd-ad61-367310a2686a

**Published:** 2011-07-12

**Authors:** Thore Santel, Gabi Pflug, Nasr Y. A. Hemdan, Angelika Schäfer, Marcus Hollenbach, Martin Buchold, Anja Hintersdorf, Inge Lindner, Andreas Otto, Marina Bigl, Ilka Oerlecke, Antje Hutschenreuter, Ulrich Sack, Klaus Huse, Marco Groth, Claudia Birkemeyer, Wolfgang Schellenberger, Rolf Gebhardt, Mathias Platzer, Thomas Weiss, Mookambeswaran A. Vijayalakshmi, Monika Krüger, Gerd Birkenmeier

The 12th author's last name is misspelled. The correct author name is: Antje Hutschenreuther. 

